# Dengue Encephalitis: A Case Series on a Rare Presentation of Dengue Fever

**DOI:** 10.7759/cureus.21615

**Published:** 2022-01-25

**Authors:** Samiksha Gupta, Gautam Jesrani, Yuvraj S Cheema, Vivek Kumar, Aman Garg

**Affiliations:** 1 Internal Medicine, Government Medical College and Hospital, Chandigarh, Chandigarh, IND

**Keywords:** hemorrhagic encephalitis, seizures, dengue encephalitis, neurological manifestation, dengue fever

## Abstract

Neurological manifestations like encephalitis, especially hemorrhagic encephalitis, are rarely described in dengue fever (DF), and the gamut may affect any part of the central or peripheral nervous system. Herein, we report two cases from Northern India, presenting with fever and altered sensorium, subsequently diagnosed with DF. Imaging studies revealed hemorrhagic encephalitis in both of them but one of them had a grave outcome, unfolding the fatal nature of the disease. The report enlightens DF as an unusual etiology of encephalitis and the importance of considering the infirmity as a differential in patients with neurological manifestations.

## Introduction

Dengue virus (serotypes DEN-1, 2, 3, 4) is a Flavivirus, which is transmitted through the bite of an infected *Aedes agypti* mosquito, and as per the census of the World Health Organization (WHO), the number of dengue cases has increased to eight times in the last two decades [1]. DEN-2 and DEN-3 are the serotypes traditionally involved in neurological complications [2]. The virus can affect any part of the nervous system, including the peripheral nervous system (PNS) and ophthalmic structures, due to its neurotropic nature. The major identifiable risk factors contributing to neurological involvement are high-grade fever, thrombocytopenia, elevated hematocrit, and hepatic dysfunction [3].

## Case presentation

Case 1

A 37-year-old female homemaker, with no comorbidities, presented to the emergency department with altered mentation. There was a history of moderate grade, intermittent fever, and associated chills and rigor of five days’ duration, along with a diffuse, dull, aching, continuous headache of three days’ duration and three to four episodes of non-bilious vomiting prior to the presentation. There was no pain in the abdomen, diarrhea, or seizures. On examination, she was febrile (102°F) but had a regular pulse rate of 102 beats/min, blood pressure 100/80 mmHg, Glasgow coma scale (GCS) 8/15 (eye-opening: 2/4, verbal response: 3/5, motor response: 3/6) and bilaterally reactive pupils. Meningeal signs were absent with bilateral flexor plantar response and no focal deficit. The rest of the systemic examination was normal.

**Figure 1:**
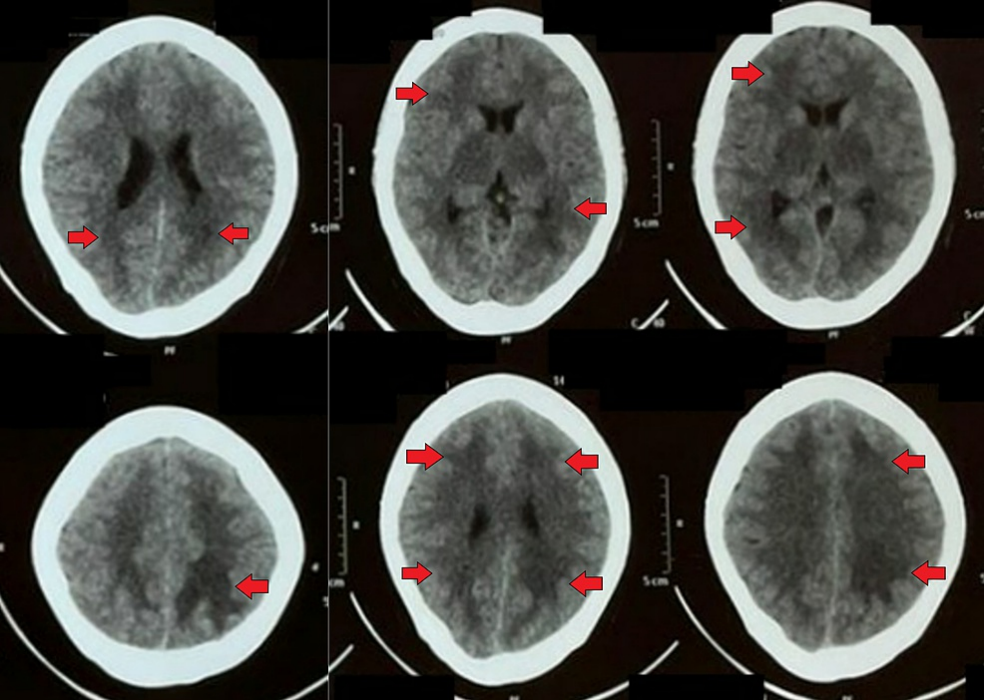
Non-contrast computed tomography of the brain demonstrating diffuse cerebral edema (red arrows)

She was then subjected to non-contrast computed tomography (NCCT) of the brain, which suggested diffuse cerebral edema (Figure 1).

On investigations, the patient had thrombocytopenia and transaminitis (Table 1). Cerebrospinal fluid (CSF) analysis had normal protein and glucose levels, no pleocytosis, and an absence of oligoclonal bands. On Gram staining and culture of the CSF, no organism was isolated and herpes simplex virus polymerase chain reaction (HSV PCR) was reported negative. She was managed on the lines of a presumptive diagnosis of infective encephalitis and started on intravenous ceftriaxone 2 gm and dexamethasone 8 mg 12 hourly.

**Table 1:** Laboratory investigations of Case 1 ALP, alkaline phosphatase; serum glutamic oxaloacetic transaminase, SGOT; serum glutamic pyruvic transaminase, SGPT; immunoglobulin, Ig

INVESTIGATION	Value	Normal range
Hemoglobin (g/dL)	13.1	13-16
Platelets (×10^9^/L)	76	150-400
Total leukocyte count (×10^9^/L)	5.2	4-12
Bilirubin (mg/dL)	1.0	0.2-1
ALP (IU/L)	88	30-150
SGOT (IU/L)	409	10-40
SGPT (IU/L)	225	10-40
Albumin (gm/dL)	4.3	3.5-5.5
Sodium (mmol/L)	141	135-145
Potassium (mmol/L)	3.9	3.5-5.5
Urea (mg/dL)	31	15-40
Creatinine (mg/dL)	0.7	<1.3
Dengue IgM serology	Positive	-
Scrub typhus serology	Negative	-
Malaria antigen	Negative	-
Leptospira IgM serology	Negative	-
Cytomegalovirus IgM serology	Negative	-
Varicella-specific IgM serology	Negative	-
Epstein-Barr virus IgM serology	Negative	-

**Figure 2:**
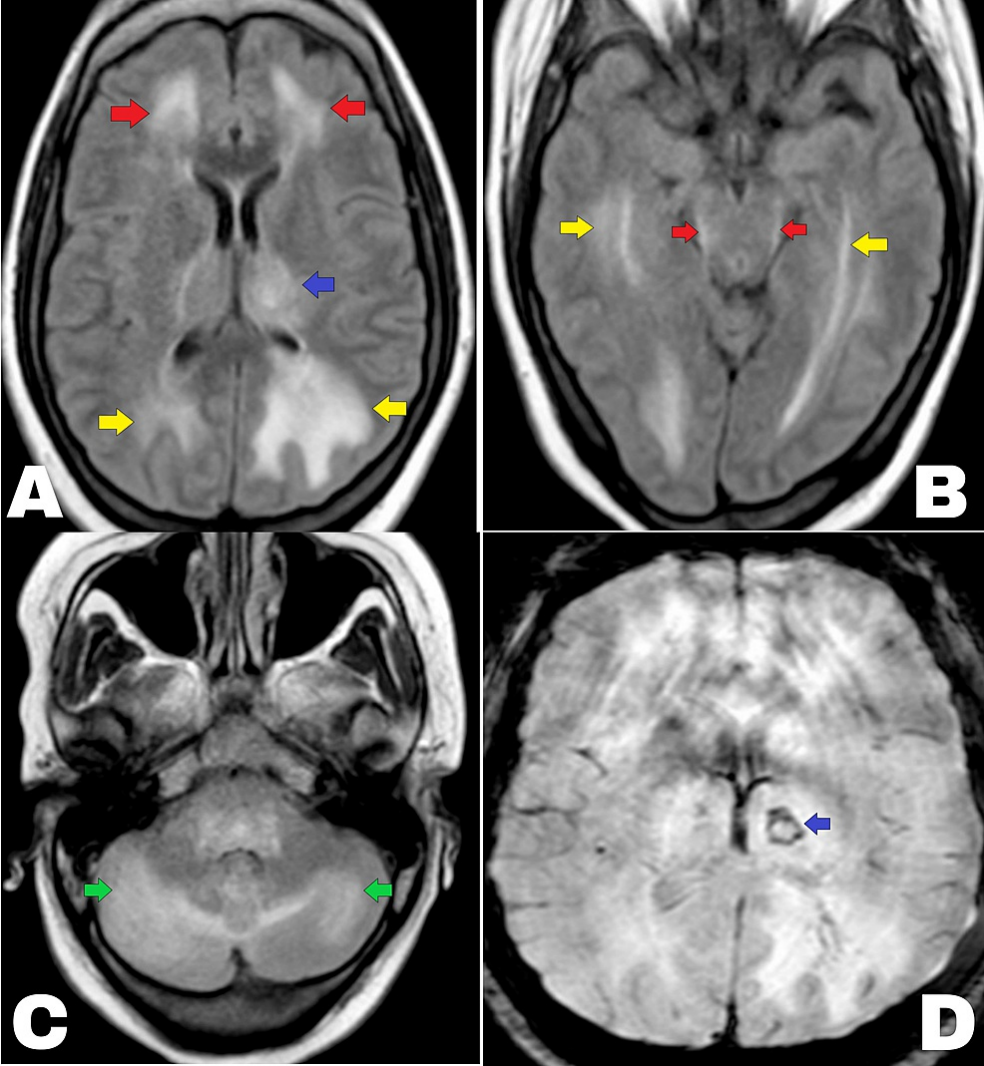
Magnetic resonance imaging (fluid-attenuated inversion recovery imaging) demonstrating hyperintensities in bilateral cerebral [frontal lobes (red arrow) and left thalamus (blue arrow) and occipital lobes (yellow arrow) in A], midbrain (red arrow) and temporal lobes (yellow arrow) in B, and cerebellar hemispheres (green arrow in C). Susceptibility-weighted imaging (D) suggested thalamic bleed (blue arrow).

In view of thrombocytopenia and the ongoing endemic of tropical illnesses, the patient was investigated for malaria, dengue fever, scrub typhus, enteric fever, and leptospirosis. Later, contrast-enhanced magnetic resonance imaging (CEMRI) of the brain revealed hyperintensities in bilateral cerebral (frontal and temporal lobes) and cerebellar hemispheres, midbrain, and thalami (Figure 2). Susceptibility-weighted imaging suggested thalamic bleed (Figure 2) and all these features were suggestive of acute hemorrhagic necrotizing encephalitis. Angiography of the cerebral vessels was normal. Surprisingly, the patient came out to be Dengue NS-1 antigen positive by enzyme-linked immunoassay sorbent assay (ELISA), and from Day 3 onwards, she became afebrile with an improvement in GCS 13/15 (Eye-opening: 4/4, verbal response: 4/5, motor response: 5/6) and platelet count. Her antibiotic was stopped after three days and steroids were tapered but, unfortunately, she developed bilateral horizontal gaze palsy, dysarthria, and truncal ataxia. However, repeat NCCT head had no worsening or any new lesions. On the seventh day, the patient was discharged with a GCS of 15/15 and resolving transaminitis. Follow-up at one month demonstrated minimal improvement in her residual symptoms.

Case 2

A 27-year-old male, with no history of addictions or co-morbidities, presented in poor sensorium, after three episodes of generalized tonic-clonic seizures in six hours’ duration. There was a history of high-grade fever and associated chills and rigor for four days and persistent non-bilious vomiting for two days. No prior history of concurrent headache, motor weakness, or similar past episodes was documented. On presentation, he was febrile (103°F) and had a pulse rate of 115 beats/min, blood pressure 98/70 mmHg, GCS 10/15 (Eye-opening: 4/4, verbal response: 2/5, motor response: 4/6), bilaterally reactive pupils, and absent meningeal signs. The other system examinations were unremarkable. So, he was started on anti-epileptics and an intravenous antibiotic (ceftriaxone 2 gm twice a day) initially.

**Figure 3:**
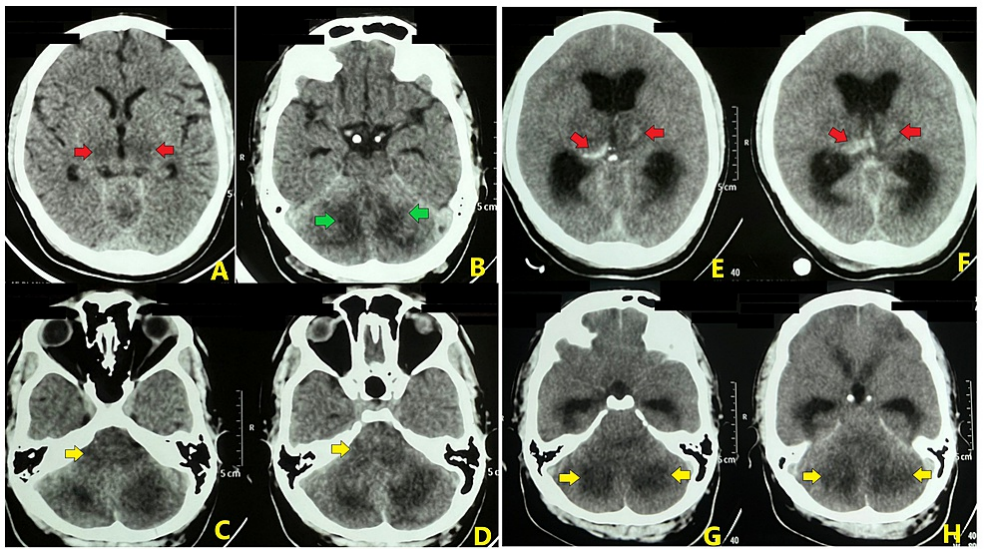
Non-contrast computed tomography of the brain demonstrating hypodensity in bilateral thalami (red arrow in A), bilateral cerebellar hemisphere (green arrow in B), and medulla (yellow arrow in C and D). Next computed tomography demonstrated intrathalamic hemorrhage (red arrow in E and F) with diffuse cerebral edema hydrocephalus and diffuse hypodensities in the cerebellar hemisphere (yellow arrow in G and H).

His first NCCT brain indicated ill-defined hypodensity in bilateral thalami, bilateral cerebellar hemisphere, and medulla (Figure 3). Also, investigations suggested thrombocytopenia and deranged hepatic enzymes (Table 2).

**Table 2:** Laboratory investigations of Case 2 ALP, alkaline phosphatase; serum glutamic oxaloacetic transaminase, SGOT; serum glutamic pyruvic transaminase, SGPT; immunoglobulin, Ig

INVESTIGATION	Value	Normal range
Hemoglobin (g/dL)	15.1	13-16
Platelets (×10^9^/L)	15	150-400
Total leukocyte count (×10^9^/L)	4.2	4-12
Bilirubin (mg/dL)	0.9	0.2-1
ALP (IU/L)	70	30-150
SGOT (IU/L)	1392	10-40
SGPT (IU/L)	813	10-40
Albumin (gm/dL)	3.9	3.5-5.5
Sodium (mmol/L)	137	135-145
Potassium (mmol/L)	3.6	3.5-5.5
Urea (mg/dL)	28	15-40
Creatinine (mg/dL)	0.8	<1.3
Hepatitis A serology	Negative	-
Hepatitis E serology	Negative	-
Hepatitis B serology	Negative	-
Hepatitis C serology	Negative	-
Dengue IgM serology	Positive	-
Scrub typhus serology	Negative	-
Malaria antigen	Negative	-
Leptospira IgM serology	Negative	-
Cytomegalovirus IgM serology	Negative	-
Varicella-specific IgM serology	Negative	-
Epstein-Barr virus IgM serology	Negative	-

CSF analysis was deferred due to thrombocytopenia, and single donor platelets were transfused for the same. The patient had no seizure thereafter and platelet count improved to 56,000 × 10^9^/L on the next day, so a lumbar puncture was performed. CSF analysis revealed normal protein and glucose levels, no pleocytosis, and an absence of oligoclonal bands. HSV PCR was reported negative, and serology for toxoplasma was inconclusive in CSF. Since there was no improvement in sensorium, CEMRI brain was planned, but his GCS deteriorated to 4/15 with bilaterally dilated pupils on the next day. The patient was intubated immediately and shifted to the intensive care unit. Repeat NCCT brain was done, which revealed diffuse cerebral and cerebellar edema, with compression of the fourth ventricle causing hydrocephalus and diffuse hypodensity in bilateral thalami and cerebellar hemisphere, with intrathalamic hemorrhage (Figure 3). Anti-edema measures were started [head end elevation, intravenous mannitol (0.5 mg/kg, 8 hourly), and dexamethasone (4 mg 8 hourly)]. However, the patient eventually succumbed to the illness four hours after the intubation. Post-obituary reports demonstrated positive Dengue NS-1 antigen by ELISA, negative serology for hepatitis A, E, B, and C, malaria, scrub typhus, typhoid fever, and leptospirosis. CSF culture did not reveal the growth of any organism and Indian ink preparation for cryptococcus was negative.

## Discussion

As per the WHO 2009 classification, dengue encephalitis is categorized as severe dengue. Central nervous system (CNS) involvement (ischemic/hemorrhagic stroke, encephalitis, acute disseminated encephalomyelitis, and transverse myelitis), PNS manifestations (long thoracic nerve palsy, abducens nerve palsy, facial palsy, brachial neuritis, myositis, hypokalaemic paralysis, and Guillain-Barre syndrome), and ophthalmic complications (maculopathy, optic neuropathy, and subconjunctival and vitreous hemorrhage) have been described in the context of DF [4-9].

Encephalitis is inflammation of the brain parenchyma, often due to viral infection. Dengue virus was earlier considered non-neurotropic, but recent observations have proven this wrong. Direct viral invasion in the CNS, possibly as a result of the blood brain barrier disruption, is the proposed pathogenesis for neural involvement. Additionally, autoimmune reactions and metabolic variations have also been established, which further worsen neurological infirmity [10-11].

Three to seven days is the usual interval between the onset of neurological symptoms and systemic features of DF. Headache, fever, altered sensorium, seizures, and focal neurological deficit, in the absence of any metabolic abnormalities, are the typical features of encephalitis. Thrombocytopenia is depicted in the hemogram, and CSF analysis reveals pleocytosis with viral growth on culture. Still, CSF with normal cellularity can be seen in 75% of cases of dengue encephalitis [12].

The majority of the patients with dengue encephalitis have normal findings on neuroimaging [13]. MRI is preferred over CT head, although the findings are often non-specific [13]. The commonly affected sites of CNS are the thalamus and basal ganglia, followed by the cerebral cortex and cerebellar hemispheres. Findings such as focal hemorrhage, patchy areas of diffusion restriction, and post-contrast enhancement have also been defined.

Adequate hydration with intravenous fluids, antipyretics for fever, seizures control with anti-epileptic drugs, and transfusion of blood products (if required) forms the fundamental treatment in DF. Raised intracranial pressure can be treated with head elevation, mannitol, and steroids, with regular scrutinization of consciousness level. Our patients were managed similarly; nevertheless, the patient in Case 2 succumbed to his illness. The prognosis in dengue encephalitis is good, although mortality can range up to 3.7% [14].

## Conclusions

Dengue encephalitis should be kept as a differential in patients with a short history of fever and altered sensorium in countries where the DF is endemic, especially in the post-monsoon season. Thrombocytopenia is generally a clue for tropical illnesses like dengue, malaria, and scrub typhus and can be used as a basis for the evaluation of these infirmities. Also, physicians should have a high index of clinical suspicion since the prognosis is good if managed on time.
